# New concepts in EBV-associated B, T, and NK cell lymphoproliferative disorders

**DOI:** 10.1007/s00428-022-03414-4

**Published:** 2022-10-11

**Authors:** Leticia Quintanilla-Martinez, Steven H Swerdlow, Thomas Tousseyn, Carlos Barrionuevo, Shigeo Nakamura, Elaine S. Jaffe

**Affiliations:** 1 Institute of Pathology and Neuropathology and Comprehensive Cancer Center Tübingen, University Hospital Tübingen, Eberhard-Karls-University, Tübingen, Germany; 2 Department of Pathology, University of Pittsburgh School of Medicine, Pittsburgh, PA USA; 3 Department of Pathology, UZ Leuven, University Hospitals, Leuven, Belgium; 4 Department of Pathology, Instituto Nacional de Enfermedades Neoplásicas, Lima, Perú; 5 Department of Pathology, Nagoya University Hospital, Nagoya, Japan; 6 Laboratory of Pathology, Center for Cancer Research, National Cancer Institute, National Institutes of Health, Bethesda, MA USA

**Keywords:** EBV+ lymphoproliferations, Mucocutaneous ulcer, PTLD, EBV+ LPD in children, Immunosuppression

## Abstract

EBV-associated lymphoproliferative disorders (LPD) include conditions of B, T, and NK cell derivation with a wide clinicopathological spectrum ranging from indolent, self-limiting, and localized conditions to highly aggressive lymphomas. Since the 2016 World Health Organization (WHO) lymphoma classification, progress has been made in understanding the biology of the EBV-associated LPDs. The diagnostic criteria of EBV+ mucocutaneous ulcer and lymphomatoid granulomatosis have been refined, and a new category of EBV-positive polymorphic B cell LPD was introduced to encompass the full spectrum of EBV-driven B cell disorders. The differential diagnosis of these conditions is challenging. This report will present criteria to assist the pathologist in diagnosis. Within the group of EBV-associated T and NK cell lymphomas, a new provisional entity is recognized, namely, *primary nodal EBV+ T or NK cell lymphoma*. The EBV + T and NK cell LPDs in children have undergone major revisions. In contrast to the 2016 WHO classification, now four major distinct groups are recognized: hydroa vacciniforme (HV) LPD, severe mosquito bite allergy, chronic active EBV (CAEBV) disease, and systemic EBV-positive T cell lymphoma of childhood. Two forms of HV LPD are recognized: the classic and the systemic forms with different epidemiology, clinical presentation, and prognosis. The subclassification of PTLD, not all of which are EBV-positive, remains unaltered from the 2016 WHO classification. This review article summarizes the conclusions and the recommendations of the Clinical Advisory Committee (CAC), which are summarized in the International Consensus Classification of Mature Lymphoid Neoplasms.

## Introduction

Since the updated 2016 World Health Organization (WHO) lymphoma classification, [1] progress has been made in understanding the biology of the EBV-associated LPDs [2]. Discussions at the Clinical Advisory Committee (CAC) for the classification of mature lymphoid neoplasms organized by the European Association for Haematopathology (EAHP) and the Society for Hematopathology (SH) focused on areas in which new advances have occurred. The discussion and conclusions during the CAC resulted in the refinement of the diagnostic criteria for some diseases (i.e., EBV+ mucocutaneous ulcer, lymphomatoid granulomatosis), the recognition of new disease entities (i.e., primary nodal EBV+ T or NK cell lymphoma), and the introduction in the lymphoma classification of EBV-positive polymorphic B cell LPD (EBV+ polymorphic B cell LPD), NOS. The EBV + T and NK cell LPDs in children have undergone major revisions. The iatrogenic immunodeficiency-associated LPDs include posttransplant lymphomas (PTLD). It was decided to retain PTLD as a separate subgroup based mainly on major differences in clinical management. The subclassification of PTLD, not all of which are EBV-positive, remains unaltered from the 2016 WHO classification.

In this review, we will focus on those EBV-associated entities where significant knowledge has recently been acquired, resulting in changes included in the 2022 International consensus classification (2022 ICC). [3]

## EBV-positive B cell lymphoproliferative disorders

EBV-positive B cell LPDs includes disorders with a wide clinicopathological spectrum ranging from indolent, self-limiting, and localized conditions to highly aggressive lymphomas (Table 1). From a morphological point of view, all these disorders share some morphological and phenotypical features; however, the clinical context is important for the differential diagnosis (Table 2). Because of the frequent presence of Hodgkin and Reed–Sternberg (HRS)-like cells in these disorders, the differential diagnosis with EBV+ classic Hodgkin lymphoma (CHL) can be challenging; however, the expression of B cell markers in >50% of the tumor cells, extranodal presentation, and/or EBV latency III excludes the diagnosis of CHL. Extended B cell antibody panels are critical in this setting. [3, 4]

**Table 1:** EBV-positive B cell lymphoproliferative disorders

EBV-positive mucocutaneous ulcer*
EBV-positive diffuse large B cell lymphoma, NOS
Diffuse large B cell lymphoma with chronic inflammation
Fibrin-associated diffuse large B cell lymphoma
Lymphomatoid granulomatosis
EBV-positive polymorphic B cell lymphoproliferative disorder, NOS*

^*^Changes from the 2016 WHO classification

**Table 2:** Comparison among the different B cell EBV-associated lymphoproliferative disorders

Disease	Clinical features	Pathologic features	EBV viral load and EBV latency	Pathogenesis	Molecular findings
EBV+ mucocutaneous ulcer	-Mostly elderly patients -Isolated well-circumscribed lesion in the oropharyngeal mucosa, skin or gastrointestinal tract -The presence of ≥2 lesions favor the diagnosis of EBV+ polymorphic B cell LPD or EBV+DLBCL -No lymphadenopathy or organomegaly, no LDH elevation -Most cases regress spontaneously or with reduction of immunosuppression	-Polymorphous infiltrate often with immunoblasts and HRS-like cells -Rim of CD3+T cells at the base of the ulcer -Angioinvasion might be present -Wide range of EBER+ cells (small and large) might be seen§ -Large cells show B cell phenotype with CD30+ and CD15+ (50%)	-The EBV viral load is negative. Important feature for the diff dx with other EBV+ LPDs EBV latency II (50%) EBV latency III (50%)	-In elderly people immune senescence -In younger people immunosuppression (mostly iatrogenic, solid organ transplantation, primary immunodeficiency) Methotrexate, azathioprine, cyclosporin, Imatinib, and others	-B cell clonality is demonstrated in up to 50% of the cases -Restricted TCR gene rearrangement patterns
EBV+ polymorphic B cell LPD	-EBV+ LPD with and without known immunodeficiency -Extranodal involvement frequent mostly in CNS, skin, gastrointestinal tract, lung. Diff Dx with LYG -Lymph node and bone marrow involvement uncommon - Most lesions regress upon restoration of the immune system	-Lymph node with distorted architecture by a polymorphic B cell infiltrate with full range of maturation from small B cells to immunoblasts and HRS-like cells -Extranodal with destructive masses - Not angiocentric or angioinvasive -Coagulative necrosis might be present - Large cells show B cell phenotype often with CD30+ but rarely CD15+ -Wide range of EBER+ cells (small and large)§	-EBV viral load often significantly elevated -EBV latency II or III	Occurs in different clinical settings (posttransplant, iatrogenic, autoimmune disorders, primary immunodeficiency, immune senescence)	-B cell clonality is demonstrated in 50–70% of the cases -Genetic alterations are rare
Lymphomatoid granulomatosis	-Pulmonary involvement is required for the diagnosis -Extranodal involvement frequent, most commonly CNS and skin. Diff dx with EBV+ polymorphic B cell LPD -Lymph node and bone marrow involvement rare - Treatment with immune modulation and immunochemotherapy	-Rare angiocentric and angiodestructive EBV+LPD -EBV+ atypical cells in a T cell rich background -various degrees of coagulative necrosis -Large cells show B cell phenotype, CD30+ and negative for CD15 -Three grades are recognized based on the number of EBV+B cells	-EBV viral load often low or negative -EBV latency predominantly II -EBV latency III should prompt to investigate an underlying immunosuppression	-Defective immune surveillance despite lack of known immunodeficiency -Dysfunctional CD8 cytotoxic cells	-LYG grades 2 and 3 usually show B cell clonality -TCR gene rearrangements are not present. Restricted patterns might be observed
EBV+ DLBCL	-Occurs in a wide age range -Immunocompetent patients -Elderly patients present common in extranodal sites (40%) as well as nodal involvement. Associated with poor prognosis -Younger patients (<45 years) mainly a nodal disease and better prognosis	- >80% of viable tumor cells EBER+ - A THRLBCL pattern is frequently observed in younger patients associated with better prognosis -Often HRS-like cells show B cell phenotype, CD30+, and rarely CD15+ -Often non-GC phenotype (MUM1+, CD10-) -PD-L1 and PD-L2 often express in the younger patients (95% vs 11%)	-EBV viral load often significantly elevated -EBV latency II predominates EBV latency III (7-12%) -EBV latency III should prompt to investigate an underlying immunosuppression	-In elderly patients, immune senescence -In younger patients an inhibitory, tolerogenic immune environment	-B cell clonality is demonstrated in most cases -Restricted TCR gene rearrangement patterns Oligoclonal (60%), rarely monoclonal -Recurrent alterations in NF-kB, WNT and IL6/JAK/STAT pathways *-MYD88* and *CD79A* mutations rare

*LPD*, lymphoproliferative disorder; *EBV*, Epstein–Barr virus; *HRS-like*, Hodgkin–Reed–Sternberg-like; *DLBCL*, diffuse large B cell lymphoma; *diff dx*, differential diagnosis

*THRLBCL*, T cell histiocyte-rich large B cell lymphoma. *LYG*, lymphomatoid granulomatosis. *TCR*, T cell receptor

^§^, wide range of EBER+ B cells is an important feature for the differential diagnosis with classic Hodgkin lymphoma

## EBV-positive mucocutaneous ulcer

EBV-positive mucocutaneous ulcer (EBV+MCU) was introduced in the 2016 WHO classification as a provisional entity [1] but is now considered a definite entity [3]. During the CAC discussions, there was some uncertainty as whether the presence of multiple lesions is acceptable for a diagnosis of EBV+MCU. The consensus criteria stress that these are solitary lesions mainly in the oropharyngeal region with usually a self-limited, indolent course [5, 6]. Cutaneous and gastrointestinal (GI) presentations are frequently associated with iatrogenic immunosuppression [7, 8]. In cases with ≥2 skin or GI lesions, the term EBV+ polymorphic B cell LPD or when appropriate, EBV-positive diffuse large B cell lymphoma (EBV+ DLBCL), not otherwise specified (NOS) or other specific type of EBV-positive lymphoma/LPD is preferred. [3]

### Clinical features

EBV+MCU occurs in the setting of defective surveillance for EBV, due to advanced age, iatrogenic immunosuppression, solid organ transplantation, primary immunodeficiency, or HIV infection [5, 7, 9–12, 13]. The median age at presentation is 70 years. Younger patients are usually associated to immunosuppression. The most commonly implicated drug is methotrexate followed by azathioprine, cyclosporin, imatinib, and others [9]. EBV+MCU are sharply circumscribed, isolated, indurated ulcers. Apart from the symptoms related to the ulcer, patients are otherwise asymptomatic without lymphadenopathy, hepatosplenomegaly, or bone marrow (BM) involvement. EBV DNA in PB is not increase, which is a useful criterion in the differential diagnosis with EBV+DLBCL, NOS. Most EBV+MCU regress spontaneously or respond to reduction of immunosuppression. In elderly patients without known immunosuppression, rituximab as a single agent is reported to achieve excellent results. Rare cases may exhibit a relapsing and remitting course without further progression. [14]

### Morphology

**Fig. 1:**
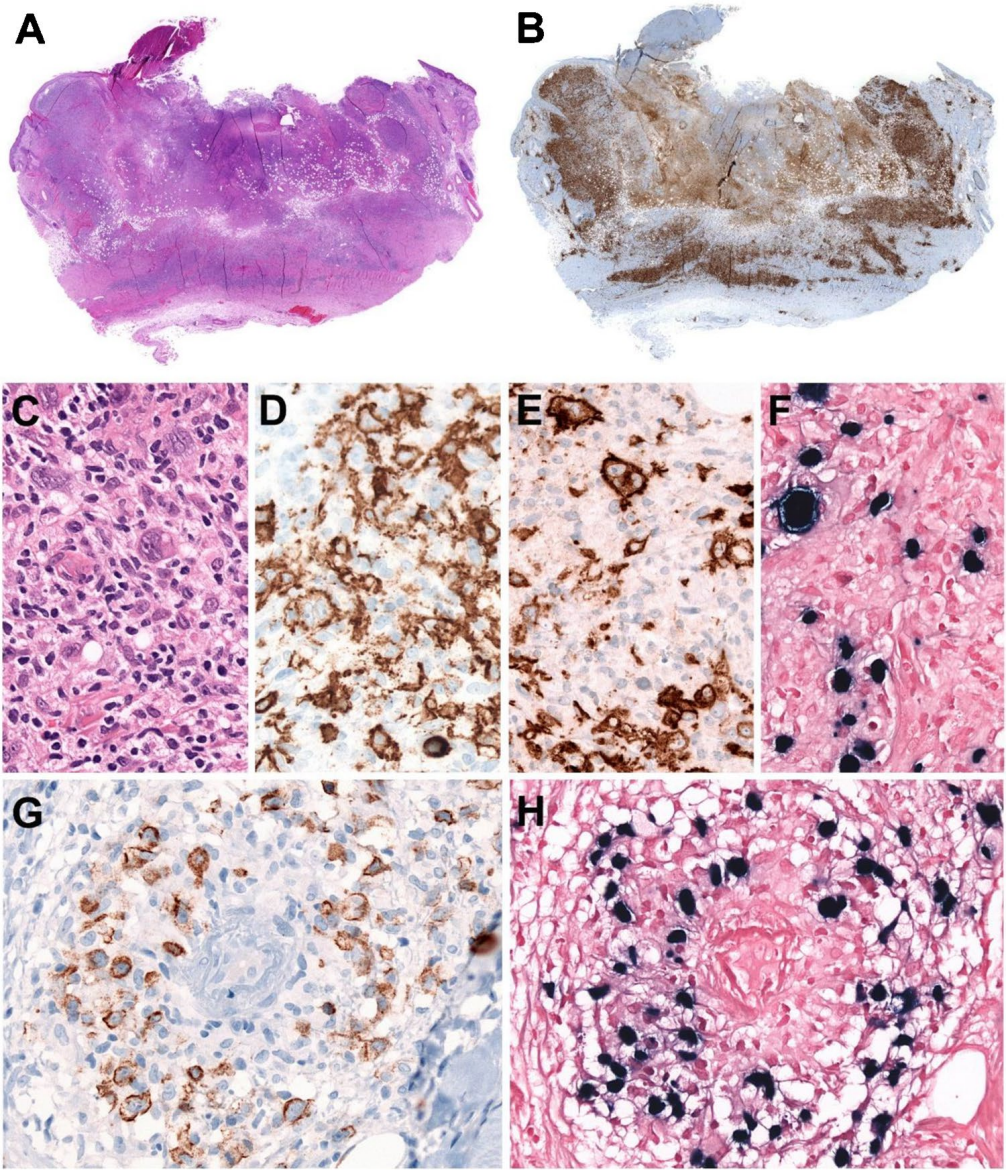
EBV-positive mucocutaneous ulcer. A solitary skin lesion in the forehead of a 70-year-old man without known immunosuppression. **A** Panoramic view of a well-circumscribed ulcer (snap-shot from scanned slide). **B** CD3 stain demonstrates abundant reactive T cells that form a rim at the base of the ulcer (snap-shot from scanned slide). **C** Higher magnification showing Hodgkin and Reed–Sternberg (HRS)-like cells in a polymorphic background. **D** CD20 stain is positive in the large HRS-like cells and in medium and small B cells. **E** CD30 stain is strongly positive in the HRS-like cells. **F** EBER in situ hybridization is positive in the HRS-like cells. Note the wide range of cells positive for EBER. **G** LMP1 is positive in B cells surrounding a blood vessel (angiocentric lesion). **H** The LMP1-positive cells are also positive for EBER. (**C**–**H**, original magnification, 400×)

A deep excisional biopsy is recommended to appreciate the characteristic architectural features of the ulcer. Histologically, the ulcers are sharply demarcated from the underlying tissue and do not display infiltrative spread (Figure 1A) [5, 15]. The infiltrate underlying the ulcer is polymorphic composed of plasma cells, histiocytes, and eosinophils, and the base of the lesion is sharply defined by a rim of abundant reactive T cells (Figure 1B). Scattered large transformed atypical immunoblasts with HRS-like cell morphology are frequently seen (Figure 1C). These cells are usually CD20+, PAX5+, OCT2+, MUM1+, and CD10- indicating a non-germinal center phenotype (Figure 1D). CD30 is positive, and CD15 is co-expressed in about half of the cases (Figure 1E) [9]. EBER might be positive mainly in the large HRS-like cells; however, in many cases, it is observed in a wide range of cells including small lymphocytes (Figure 1F). The characterization of EBV latency reveals latency II or III. EBV latency III excludes CHL and support the diagnosis of EBV+MCU. Focal necrosis is common, often with evidence of vascular damage (Figure 1G-H).

### Pathogenesis and molecular findings

Reduced CD8+ cytotoxic T cell repertoire and T cell functionality play a pathogenetic role as a result of either immunosuppression or immunosenescence [16]. EBV+MCU often involves sites with pre-existing inflammatory lesions due to various causes [8]. It is believed that in the context of tissue damage, necrosis, and fibrinous exudate, an immune sequestered environment develops resulting in a circumscribed EBV+ B cell LPD [6]. B cell receptor (BCR) gene rearrangements are detectable in less than 50% of the cases. Oligoclonal or restricted patterns of T cell receptor (TCR) gene rearrangements are often seen indicating a compromised immune surveillance. [5, 9]

## EBV-positive diffuse large B cell lymphoma, NOS

EBV+ DLBCL, NOS, is an EBV+ monoclonal B cell lymphoma [1, 17]. By definition >80% of the viable malignant cells should express EBER [18, 19, 20]. Excluded from this category are other well-defined EBV-associated lymphoproliferations (i.e., lymphomatoid granulomatosis, EBV+MCU), evidence of acute or recent EBV infection and patients with underlying immune deficiency or history or previous lymphoma or solid tumors.

### Clinical features

EBV+DLBCL, NOS, is more prevalent in Africa, Asia, and Latin America. [17, 19, 21]. It can present over a wide age range, although the disease usually occurs in individuals >50 years and often in extranodal sites (40%) [18, 19, 20]. Patients younger than 45 years present mostly with nodal disease and have better prognosis [17, 20, 22]. The clinical presentation is variable with >50% presenting with advance stage disease. Most patients have detectable EBV DNA in PB. [23]

### Morphology

**Fig. 2:**
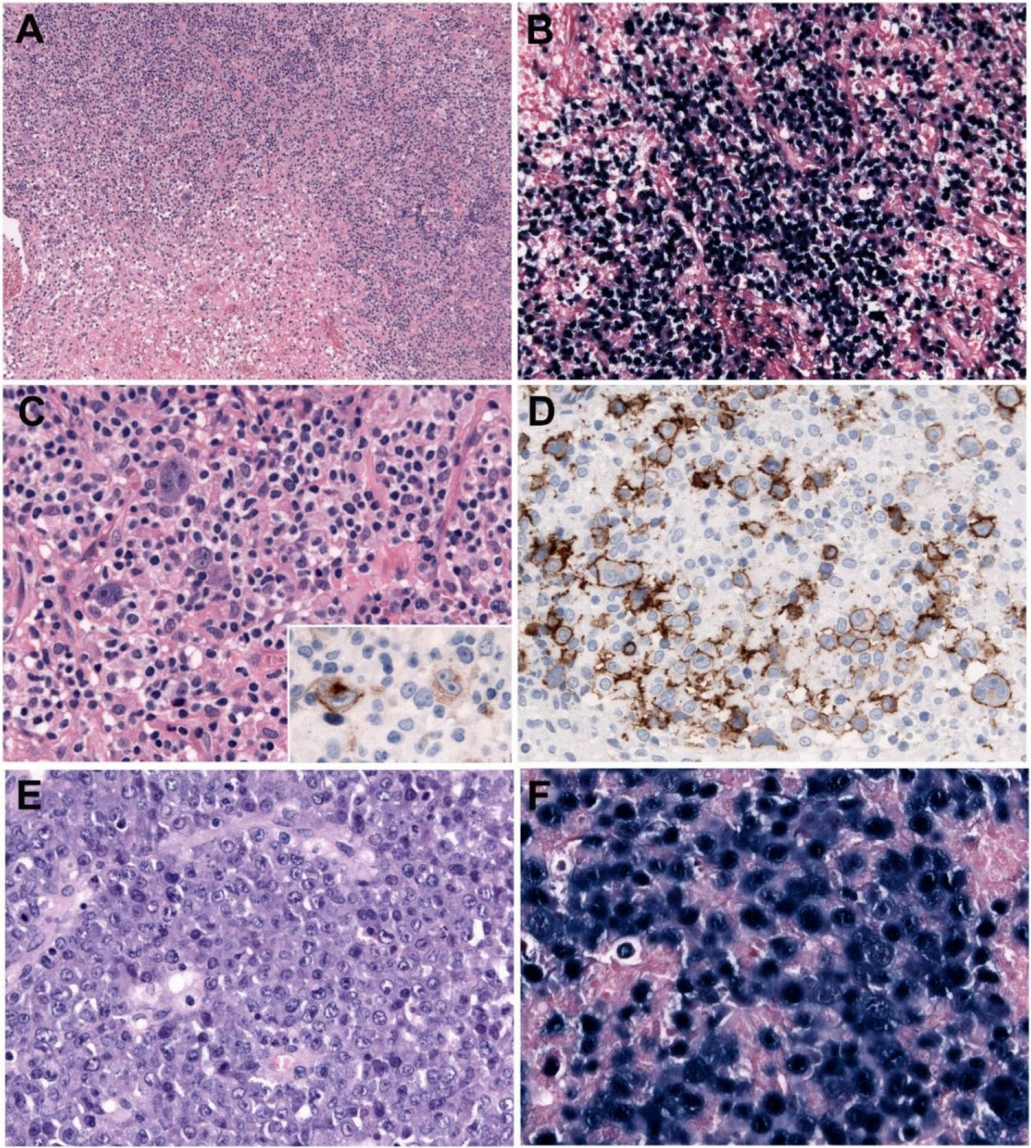
EBV-positive diffuses large B cell lymphoma, NOS. **A**–**D** Polymorphic variant with numerous HRS-like cells. **A** Low magnification of a lymph node diffusely infiltrates with geographic areas of necrosis (original magnification, ×50). **B** EBER in situ hybridization shows diffuse EBV-positive tumor cells surrounding the necrosis (original magnification, ×100). **C** Polymorphous proliferation characterized by small lymphocytes, plasma cells, histiocytes, and scattered large transformed cells mimicking Hodgkin and Reed–Sternberg (HRS) cells. Insert: the HRS-like cells are LMP1 positive (original magnification, 400×). **D** The HRS-like cells are strongly CD20 positive. Note the polymorphic B cell infiltrate. **E**–**F** Monomorphic type of EBV-positive diffuses large B cell lymphoma, NOS. **E** The lymph node is diffusely replaced by a monomorphic infiltrate of large centroblastic cells. **F** EBER in situ hybridization is positive in almost 100% of the tumor cells. (**E**–**F** original magnification, ×400)

The morphology is variable. In adults, the pattern may be monomorphic or polymorphic, but these patterns do not have prognostic impact (Figure 2A-F). In younger patients (≤45 years), a T cell-/histiocyte-rich large B cell (THRLBCL) pattern is frequently seen and associated with better prognosis [20]. The neoplastic B cells range from centroblasts, immunoblasts, or HRS-like cells embedded in a rich inflammatory background. Many cases show angiodestructive lesions with extensive coagulative geographical necrosis. Immunophenotypically, the tumor cells reveal a B cell phenotype with co-expression of CD20, CD79a, PAX5, BOB1, and OCT2 and often a non-germinal center phenotype with MUM1 expression, lack of CD10, and variable BCL6 expression [19]. Most cases are CD30+ and rarely CD15+, but other phenotypical features of CHL are lacking [16, 24]. PD-L1 and PD-L2 are often expressed in the tumor cells in young patients when compared with the elderly group (95% vs 11%), suggesting a mechanism for immune escape [20, 24]. Within the morphological spectrum in young patients, some cases show features reminiscent of CHL-nodular sclerosis but with strong expression of the B cell program and may represent rare instances of EBV+ mediastinal gray zone lymphoma [20]. LMP1 is expressed in the majority of the cases by almost all tumor cells. Although in the original description a relatively high frequency of EBNA2 expression was reported (36%, latency III) [17], in more recent series, the expression of EBNA2 has been demonstrated in 7–12% of the cases, indicating mostly an EBV latency II pattern [19, 20]. The presence of EBV latency III should prompt to investigate a source of underlying immunosuppression before assigning the case into the “NOS” category. [19]

### Pathogenesis and molecular findings

EBV+DLBCL, NOS, in older patients is believed to be related to immune senescence, whereas in younger patients, an inhibitory, tolerogenic immune environment has been proposed [20]. In most cases, clonal IGH rearrangements are detected. In addition, as a result of senescent and reduced T cell repertoire, 60% of cases show oligoclonal and rarely monoclonal TCR rearrangements [16]. In contrast to EBV-negative DLBCL, NOS, EBV+ cases show fewer driver mutations [25]. Despite the non-germinal center phenotype, these tumors rarely show *MYD88* and/or *CD79A* mutations [26]. In contrast, recurrent alterations in NF-κB, WNT, and IL6/JAK/STAT pathways are often identified. [25]

## Lymphomatoid granulomatosis

Lymphomatoid granulomatosis (LYG) is a rare angiocentric and angiodestructive EBV-driven B cell LPD admixed with numerous reactive T cells. By definition, pulmonary involvement is required for the diagnosis [3, 27]. The disease is believed to result from defective immune surveillance of EBV with most patients showing evidence of immune dysfunction, despite lack of known primary immunodeficiency. Histologically, there are significant overlapping features with other immunodeficiency-related EBV+ B cell LPDs. In the 2022 ICC, it is now emphasized that isolated CNS or GI involvement by an EBV+ lesion resembling LYG is observed usually in the context of known causes of defective immune surveillance (i.e., iatrogenic immunosuppression) showing often an EBV latency III. In this scenario, the diagnosis of LYG should not be made, and the diagnosis of EBV+ polymorphic B cell LPD or EBV+ DLBCL, NOS, should be rendered [3, 28]. The therapeutic approach of these lesions might differ.

### Clinical features

LYG presents in middle-aged adults in the fourth to sixth decade of life and has a 2:1 male predominance. The most common clinical presentation is lung involvement resulting in cough, dyspnea, and pain present in one to two thirds of the patients [15, 27]. Radiologically, there are usually multiple, variably sized bilateral cavitating nodules in mid and lower lung fields. There may be concomitant involvement of the CNS (40%), skin (34%), kidney (19%), or liver (17%). Lymph node and bone marrow involvement is extremely rare. Patients with CNS lesions develop confusion, dementia, ataxia, paresis, seizures, or cranial nerve signs. Patients with skin lesions present with multiple erythematous dermal papules and/or subcutaneous nodules resembling other causes of lobular panniculits. [29]

### Morphology

**Fig. 3:**
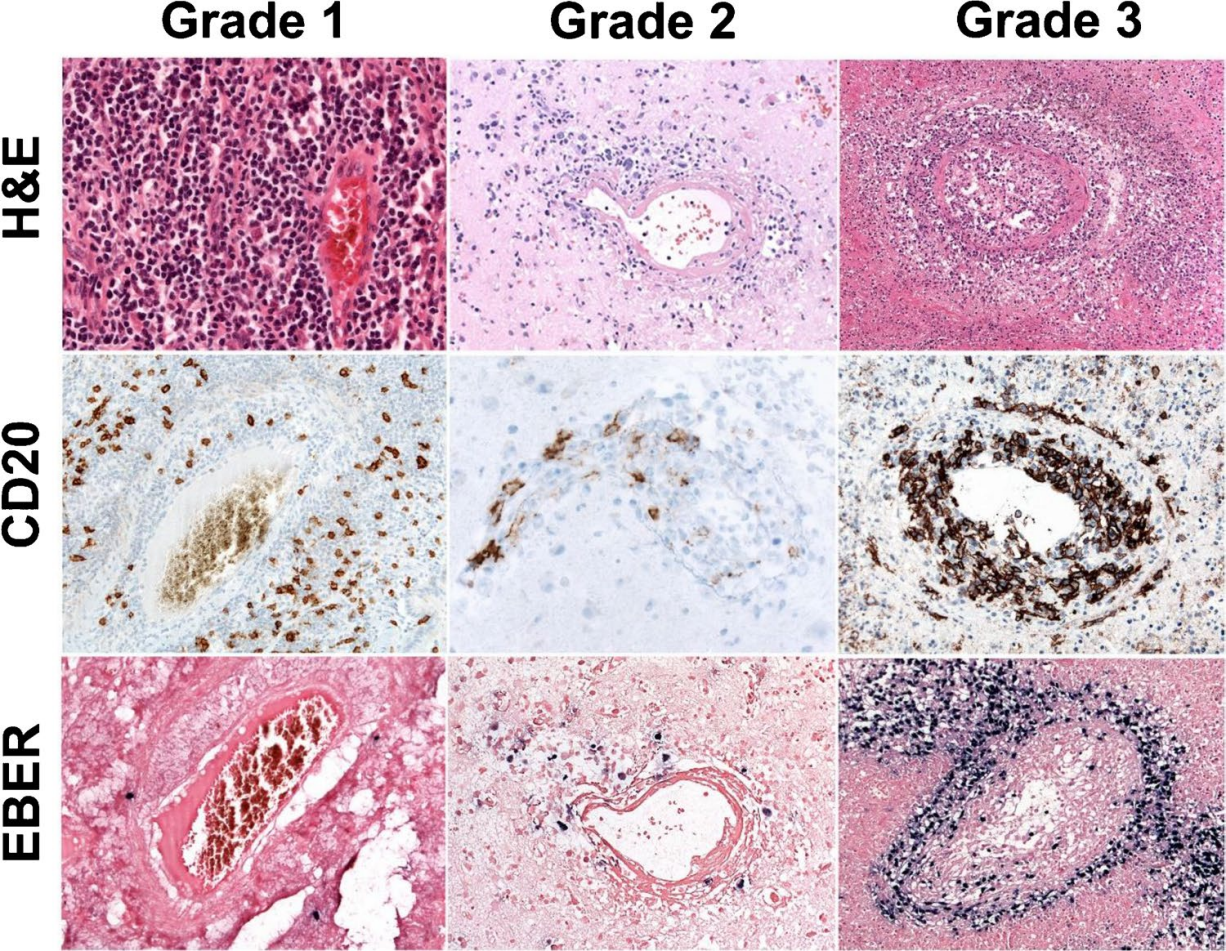
Morphologic features of lymphomatoid granulomatosis (LYG). Grade 1 is characterized by a polymorphous lymphoid infiltrate without significant atypia and lack of necrosis. <5 large EBER-positive cells in a high-power field (HPF) in an abundant reactive T cell infiltrate. (original magnification, ×200) Grade 2. Large but still sparse EBER-positive B cells (5–20/HPF) in an abundant reactive T cell infiltrate. Necrosis is more common. (Upper and lower figure, original magnification ×200; middle, ×400) Grade 3. Large EBER-positive B cells are common (up to 50/HPF) and may form aggregates and display immunoblastic or Hodgkin- and Reed–Sternberg-like morphology. Necrosis is common. (Upper and lower figure, original magnification, ×100; middle, ×200)

The disease is characterized by nodules containing a polymorphic lymphoid infiltrate with angiocentric distribution. There is a variable number of EBV+ B cells with some degree of plasmacytoid differentiation in a background of small T cell lymphocytes admixed with histiocytes, plasma cells, and immunoblasts. LYG is graded according to the number of EBV+ B cells and their degree of cytological atypia, which offers prognostic information and guidance for therapy (Figure 3).

The tumor cells are CD20+, CD30+, and negative for CD15. The small CD3+ reactive T cells are predominantly CD4+ with few CD8+ cells. EBV latency might be II or III.

Differential diagnosis with EBV+DLBCL, NOS, might be challenging and is based on the presence of larger sheets of EBV+ atypical B cells beyond the acceptable spectrum for LYG and reduced numbers of reactive T lymphocytes.

### Pathogenesis and molecular findings

LYG is hypothesized to develop in an underlying defective immune surveillance of EBV-infected B cells, particularly a functional defect in CD8+ cytotoxic T cells [27]. In LYG grades 2 and 3, IGH monoclonal rearrangement is often demonstrated, as opposed to grade 1, where clonality might not be detected. Different clonal rearrangements might be found in different lesions. TCR clonal rearrangements are not present; however, restricted patterns might be observed. [29]

## EBV-positive polymorphic B cell lymphoproliferative disorder, NOS

EBV+ polymorphic B cell LPD, NOS, is a termed used for EBV+ B cell proliferations with or without known immunodeficiency that cannot be more precisely categorized [9]. The 2022 ICC introduced this new category in the lymphoma classification [3]. By definition, the term should be reserved for cases with altered tissue architecture and a polymorphic infiltrate that does not fulfill criteria for the diagnosis of lymphoma, or there is uncertainty due to a small size or low-quality biopsy. In tissues with low or modest numbers of EBV+ B cells without distortion of the nodal architecture, the term EBV reactivation is preferred.

### Clinical features

**Fig. 4:**
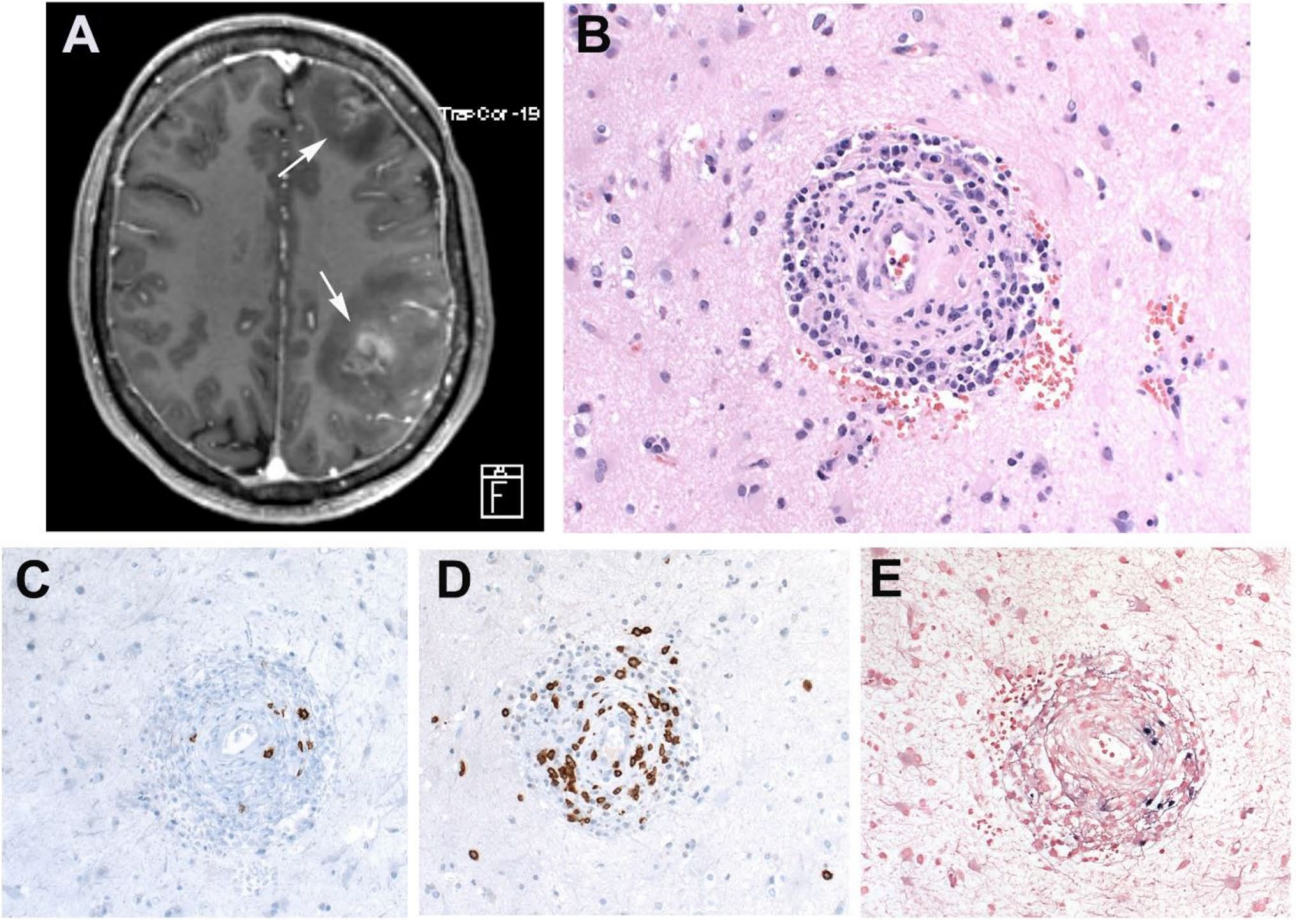
EBV-positive polymorphic B cell lymphoproliferative disorder, NOS. Patient with rheumatoid arthritis under methotrexate therapy. **A** A magnetic resonance tomography (MRT) reveals intracerebral lesions in both hemispheres (white arrows). **B** Brain biopsy showing a perivascular infiltrate mainly of small cells without atypia. **C** CD20 shows few B cells, whereas CD3-positive cells predominate (**D**). **E** EBER in situ hybridization shows few positive B cells mimicking lymphomatoid granulomatosis (LYG) grade 2. (**B**–**E**, original magnification, 400×)

**Fig. 5:**
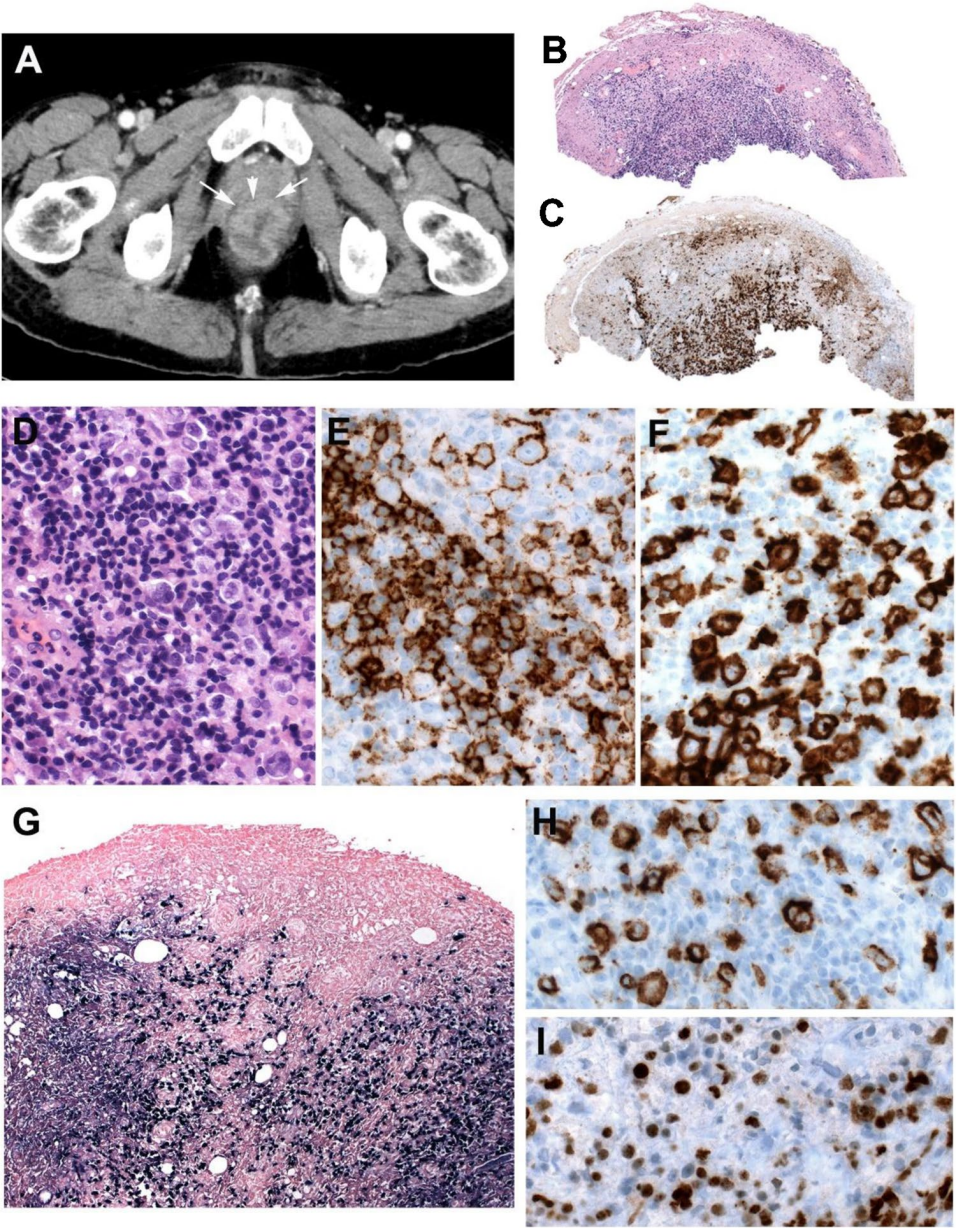
EBV-positive polymorphic B cell lymphoproliferative disorder, NOS. Patient with known Crohn’s disease under azathioprine therapy. **A** Abdominal CT scan reveals enlarged retroperitoneal lymph nodes and thickening of the rectal intestinal wall (white arrows). **B** Panoramic view of the rectal biopsy showing an ulcer with a dense polymorphic infiltrate (snap-shot from scanned slide) mimicking an EBV+ mucocutaneous ulcer. **C** CD3 demonstrates a rim of T cells at the base of the ulcer (snap-shot from scanned slide). **D** Higher magnification demonstrates a polymorphous infiltrate with Hodgkin and Reed–Sternberg (HRS)-like cells. **E** CD20 is positive in the large atypical cells, as well as in the small lymphocytes. **F** CD30 is positive in the B cell infiltrate. **G** Low-power view of the ulcer with abundant EBER-positive cells (original magnification, ×50). The cells are LMP1-positive (H) and EBNA2-positive (I) revealing an EBV latency 3. (**D**–**F** and **H**–**I** original magnification, ×400)

EBV+ polymorphic B cell LPD affect all ages. The clinical presentation ranges from isolated to generalized lymphadenopathy, as well as extranodal sites involvement, including the lung, CNS (Figure 4A-E), GI tract (Figure 5A-I), and skin. The symptomatology depends on the site of presentation; however, some patients present with extensive systemic symptoms. The treatment of these lesions varies from withdrawal of immunosuppression to single-agent rituximab or combination with other immunomodulators or immunochemotherapy.

### Morphology

The lesion shows distorted architecture with a polymorphic infiltrate composed by B cells with full range of maturation including small B cells, plasma cells, immunoblasts, and HRS-like cells admixed with a variable number of reactive T cells [9]. The immunophenotype is CD45+, CD20/CD79a+, PAX5+, CD30+, OCT2+, and BOB1 with rare cases being positive for CD15. Immunoglobulin light chain expression is often polytypic. EBER in situ hybridization is positive in all cases with variable expression of LMP1 and EBNA2 indicating either an EBV latency II or III. The most important differential diagnosis is EBV+DLBCL, NOS. The latter, in addition to architectural effacement, shows cytological atypia, and the neoplastic cells are usually monomorphic. Most cases lack the spectrum from plasma cells to immunoblasts seen in EBV+ polymorphic B cell lymphoproliferations. A polyclonal infiltrate supports the diagnosis of EBV+ polymorphic B cell LPD; however, a monoclonal population might be seen in both disorders. In difficult cases, it is necessary to take into consideration the clinical presentation and the anatomic site. A close communication with the hemato-oncologist is necessary.

### Pathogenesis and molecular findings

EBV+ polymorphic B cell LPD, NOS, occurs in different clinical settings underlying the diverse pathogenesis including posttransplant, primary immunodeficiency, iatrogenic immunodeficiency, autoimmune disorders, human immunodeficiency virus (HIV), and unknown causes, presumably immune senescence. Monoclonal IGH gene rearrangement are reported between 50 and 70% of the cases. Outside the posttransplant setting, karyotypic alterations are rare. Mutational analysis has not been performed systematically in this disorder; however, genetic alterations are rare, and therefore, the lesions are expected to regress upon restoration of a functional immune system. [30]

## EBV-positive T and NK cell lymphoproliferative diseases

EBV+ T and NK LPDs are prevalent in Asia and Native American population of Mexico, Guatemala, Peru, and Bolivia [2, 31–33, 34]. These disorders affect the pediatric population, young adults, and elderly patients. The 2022 ICC classification recognizes the EBV+ T and NK cell LPDs listed in Table 3.

**Table 3:** EBV-positive T and NK cell lymphoproliferative disorders

• EBV-positive T/NK lymphoproliferative disorders of childhood*
▪ Hydroa vacciniforme lymphoproliferative disorder*
-Classic
-Systemic
▪ Severe mosquito bite allergy
▪ Chronic active EBV disease, systemic (T and NK cell phenotype)*
▪ Systemic EBV+ T cell lymphoma of childhood
• Extranodal NK/T cell lymphoma, nasal type
• Aggressive NK cell leukemia
• Primary nodal EBV-positive T/NK cell lymphoma*

^*^Changes from the 2016 WHO classification

*Italics* means provisional entity

## EBV-positive T and NK cell lymphoproliferative disorders of childhood

EBV+T and NK LPDs of childhood is a group of uncommon disorders that affect mainly pediatric population but can also rarely occur in adults. This group of disorders has undergone a major revision in the 2022 ICC (Table 4) [3]. In the 2017 WHO classification, two major groups were recognized, namely, chronic active EBV (CAEBV) infection with a cutaneous and a systemic form and systemic EBV+ T cell lymphoma of childhood [35]. Because of new knowledge and better understanding of these disorders, the ICC 2022 recognizes now four major disorders: hydroa vacciniforme (HV) LPD, severe mosquito bite allergy, systemic chronic active EBV (CAEBV) disease, and systemic EBV-positive T cell lymphoma of childhood. The criteria and morphological features of severe mosquito bite allergy and systemic EBV+ T cell lymphoma of childhood remain the same. [35]

**Table 4:** Changes in the EBV-associated T and NK cell LPD

EBV-associated T and NK cell LPD WHO 2017	EBV-associated T and NK cell LPD 2022 international consensus classification
EBV+ T and NK cell LPD in childhood	EBV+ T and NK cell LPD in childhood
• Chronic active EBV infection -Cutaneous form	
Hydroa vacciniforme-like LPD	• Hydroa vacciniforme LPD ▪ *Classic form:* indolent, self-limited, more common in whites ▪ *Systemic form:* mild to severe disease, systemic symptoms (fever, lymphadenopathy, liver involvement), more common in Asia and Latin America. Treatment similar to CAEBV disease
Severe mosquito bite allergy	• Severe mosquito bite allergy
-Chronic active EBV infection, systemic form	• Chronic active EBV disease ▪ Systemic disease ▪ Only of T and NK cell type ▪ B cell type is excluded
• Systemic EBV+ T cell lymphoma of childhood	• Systemic EBV+ T cell lymphoma of childhood
Extranodal NK/T cell lymphoma, nasal type	Extranodal NK/T cell lymphoma, nasal type ▪ New genetic findings ▪ Intravascular EBV+ NK cell lymphoma might be a related disease
Aggressive NK cell leukemia	Aggressive NK cell leukemia ▪ Rare cases of EBV-negative are recognized, most common in non-Asians
Primary EBV+ nodal T and NK cell lymphoma, variant of PTCL, NOS	*Primary EBV+ nodal T/NK cell lymphoma* ▪ More common in elderly and/or immunodeficient patients ▪ Lack nasal involvement ▪ Characteristic genetic findings

*LPD*, lymphoproliferative disorder. *EBV*, Epstein–Barr virus; *NK*, natural killer

*PTCL, NOS*, peripheral T cell lymphoma, not otherwise specified. *Italics* means provisional entity

## Hydroa vacciniforme lymphoproliferative disorder

The concept and definition of HV LPD has considerably changed since the diagnosis was first introduced in the 2008 WHO classification as HV-like lymphoma. The term HV-like LPD was incorporated in the 2016 WHO classification [35, 36]. Recently, it was demonstrated that “classic” HV in western countries is also associated to EBV and, therefore, belongs to the same disease spectrum as cases from Asia and Latin America [37, 38]. Now, the term HV LPD is introduced to the 2022 ICC encompassing the various manifestations of the EBV-associated skin lesions. [3]

### Clinical features

HV LPD is a chronic EBV+ LPD of childhood with a broad spectrum of clinical aggressiveness and usually a long clinical course. Two clinical forms are now recognized:

- Classic: this form affects mainly white patients who present with an indolent clinical course and localized papulo-vesicular eruptions on sun-exposed skin and no systemic symptoms [2, 37]. There is seasonal variation with increased recurrence in spring and summer. Spontaneous remission during adolescence and clearing after photoprotection usually occurs. Rarely patients progress to a more aggressive (systemic) form of the disease.
- Systemic: this form affects especially Asians [33] and Hispanics [36, 39, 40]. Patients present with skin lesion in sun-exposed and non-exposed areas. During the presentation and exacerbation of the skin lesions, systemic symptoms (including fever, wasting, lymphadenopathy, and hepatosplenomegaly) may be present. The disease follows a protracted clinical course; however, as the disease progresses, there are more extensive and more severe skin lesions and systemic symptoms. Patients might respond initially to immunomodulating therapies and; however, eventually will require similar treatments as CAEBV disease. [41]

### Morphology

**Fig. 6:**
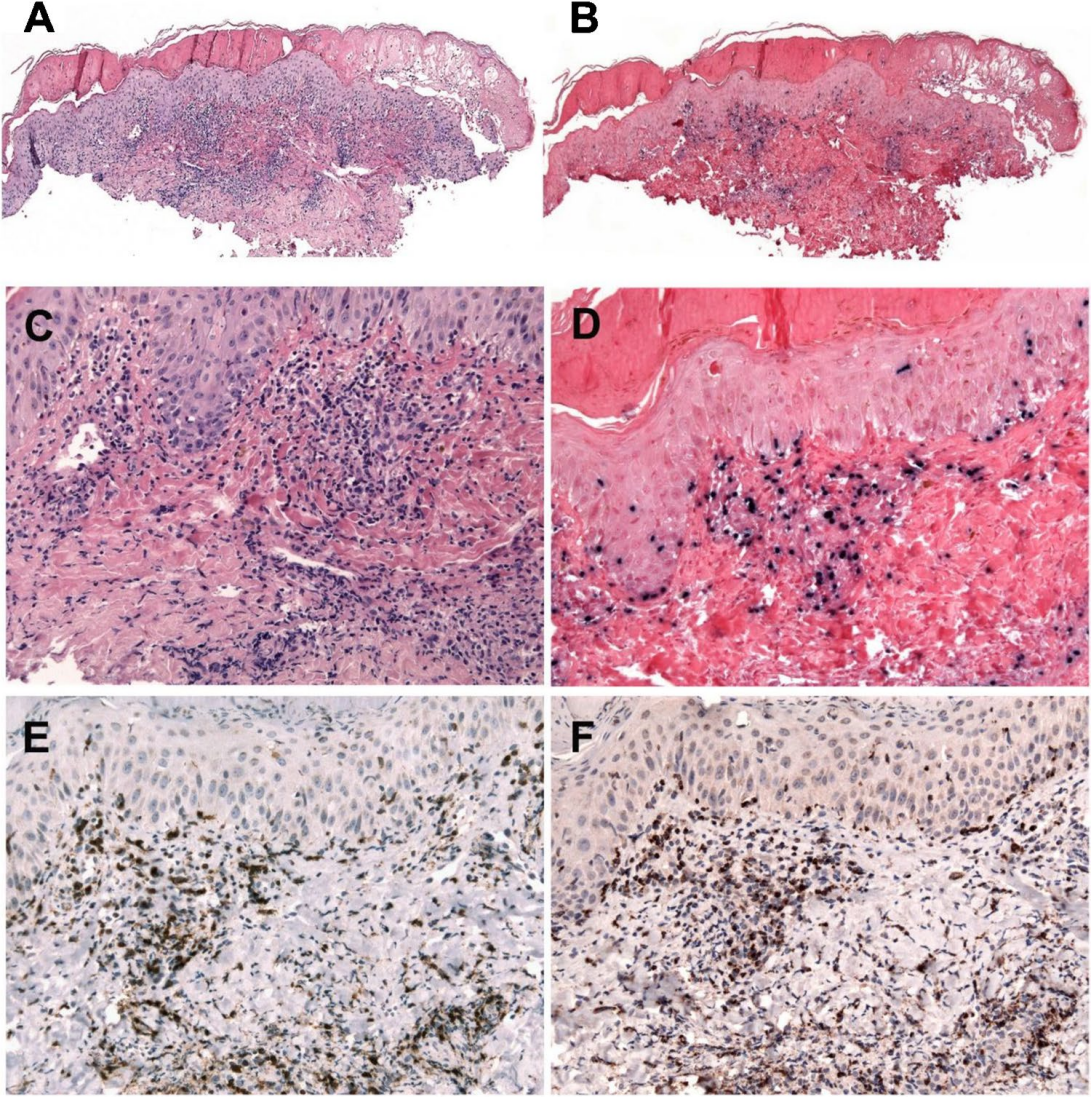
Hydroa vacciniforme lymphoproliferative disorder. **A** H&E stain of a skin biopsy with a suprabasal blister and a dense lymphoid infiltrate in the upper dermis (original magnification, ×50). **B** EBER in situ hybridization reveals the same distribution of EBER-positive cells (original magnification, ×50). **C** Higher magnification demonstrates the small- to medium-sized lymphoid cells infiltrating the upper dermis, surrounding blood vessels. **D** EBER in situ hybridization is positive in many infiltrating lymphoid cells comparable to the CD8 and TIA1 stainings shown in **E** and **F**. (**C**–**F**, original magnification, ×200)

The characteristic histological feature is epidermal reticular degeneration leading to intraepidermal spongiotic vesiculation (Figure 6A-B) The lymphoid infiltrate predominates in the dermis but may extend into the subcutaneous tissue. The infiltrate is mainly localized around adnexa and blood vessels often with angiodestructive features. The neoplastic cells are small and lack significant atypia (Figure 6C). The infiltrating cells have a cytotoxic T cell phenotype with expression of TIA-1, granzyme B, and perforin (Figure 6E-F). The cells are mostly CD8+ with few cases being CD4+ or even double CD4/CD8+ [42]. Occasional cases have an NK cell phenotype and some a mixture of T and NK cells. Cases with NK cell phenotype tend to be more panniculitic and might mimic subcutaneous panniculitis-like T cell lymphoma [36, 42, 43]. CD30 is often expressed. EBER is positive in a variable proportion of infiltrating cells (Figure 6D). LMP1 is usually negative. [36]

### Pathogenesis and molecular findings

The pathogenesis is unknown; however, genetic predisposition might play a role [37]. Most cases have clonal TCR gene rearrangements. EBV DNA is elevated in the blood, and the levels are not discriminatory between the two forms. [37]

## Chronic active EBV disease

CAEBV disease was previously referred as CAEBV infection;[35] however, since most adults are chronically, latently infected, and few acquired the disease, the term CAEBV disease is preferred [3]. CAEBV disease is a progressive disorder of ≥ 3 months in duration in which patients have markedly increased levels of EBV DNA in the blood and infiltration of organs by EBV-infected lymphocytes in the absence of known immunodeficiency. Since cases affecting B cells are usually in the context of primary immunodeficiency,[44] CAEBV disease includes now only T and NK cell disease. [3]

### Clinical features

Approximately 50% of the patients present with infectious mononucleosis-like symptoms, including fever, hepatosplenomegaly, and lymphadenopathy [33]. Other symptoms include skin rash (26%), severe mosquito bite allergy (33%), hepatitis/hepatic failure (15%), HV eruptions (10%), coronary artery aneurism (9%), diarrhea (6%), uveitis (5%), interstitial pneumonia (5%), myocarditis (4%), and intestinal perforation (11%) [45]. The clinical course varies but is usually protracted, with some patients surviving for many years without disease progression. Patients with EBV-infected T cells have a shorter survival time than patients with NK cell disease, prominent systemic symptoms, and high titers of EBV DNA in blood. In contrast, patients with NK cell disease often have severe mosquito bite allergy and high levels of IgE in serum. Hemophagocytic lymphohistiocytosis (HLH) is a life-threatening complication (24%) that usually occurs when the disease progresses. Some patients in South America present with periorbital and facial edema, high levels of EBV DNA in the blood, systemic symptoms, and EBV in internal organs, although these cases have been reported as HV LPD, in the 2022 ICC was decided to classify them as CAEBV disease due to its aggressive clinical course and lack of typical HV lesions [3, 46]. At present, hematopoietic stem cell transplantation is the only curative therapy. [41]

### Morphology

The infiltrating cells in the different organs do not show changes suggestive of a neoplastic lymphoproliferation. The diagnosis is usually made in a liver or a lymph node biopsy. The liver shows sinusoidal and portal infiltration suggestive of viral hepatitis. The lymph nodes show either follicular or paracortical hyperplasia, focal necrosis, or small granulomas. In 60% of cases, T cells are EBV-infected and in 40% NK cells. The infiltrating T cells are predominantly CD4+ with few cases being CD8+. EBER is positive. [33]

### Pathogenesis and molecular findings

The pathogenesis is unknown; however, genetic predisposition might play a role. [37]. TCR gene rearrangement might be monoclonal, oligoclonal, or polyclonal. New genetic studies suggest that some cases of CAEBV disease (especially NK cell type) carry somatic mutations in *DDX3D* and *KMT2D* indicating that it is a pre-malignant condition. The EBV genome harbors frequent intragenic deletions not found in EBV+ reactive disorders, suggesting a key role of these mutations in the development of the disease. [47]

## Extranodal NK/T cell lymphoma, nasal type

In the 2022 ICC, the definition and the clinical and morphological characteristics of extranodal NK/T cell lymphoma, nasal type (ENKTCL) remain the same as in the 2017 WHO classification [48]. The 2022 ICC retains the “nasal type” qualifier to stress the fact that cases presenting outside the nasal region have the same morphological features, namely, a tumor characterized by vascular damage and destruction, prominent necrosis, cytotoxic phenotype, and association with EBV [3]. A probably related disease is intravascular NK cell lymphoma, which is also an EBV-associated disorder and extremely rare [49]. The process is most common in skin and CNS, although other organs might be involved. It usually follows an aggressive clinical course, with median survival times ranging from 1 week to 18 months after the diagnosis. [50]

### Pathogenesis and molecular findings

The genetics of ENKTCL has been extensively investigated in recent years. Gene mutations identified in ENKTCL affect more frequently the JAK/STAT pathway (*STAT3, STAT5B, JAK3*), epigenetic regulators (*BCOR, KMT2D, ARID1A, EP300*) tumor suppressor genes (*TP53, MGA*), and the RNA helicase *DDX3X*. [31, 51, 52, 53] More recently, a large comprehensive study identified seven genetic clusters that were associated with different clinical outcomes. [54]

## Aggressive NK cell leukemia

Aggressive natural killer cell leukemia (ANKL) is a systemic NK cell neoplasm, almost always associated with EBV. Rare cases of EBV-negative ANKL have been reported [55]. EBV-negative ANKL tends to occur in older patients, mainly described in non-Asian patients, and is indistinguishable clinically and pathologically from the EBV+ cases. HLH is present in less than 50% of the cases. The clinical and morphological characteristics of ANKL remain the same as in the 2017 WHO classification. [56]

## Primary nodal EBV-positive T and NK cell lymphoma

Primary nodal EBV-positive T and NK cell lymphoma is a rare disease introduced in the 2016 WHO classification as a variant of peripheral T cell lymphoma (PTCL), NOS. New findings have led to include this lymphoma as a provisional entity in the 2022 ICC [3]. It presents more commonly in elderly and/or immunodeficient patients, lacks nasal involvement, and is more often of T rather than NK cell lineage. [57]

### Clinical features

Patients present with lymphadenopathy and systemic symptoms. By definition, there is no nasal involvement. This lymphoma is characterized by a dismal prognosis.

### Morphology

**Fig. 7:**
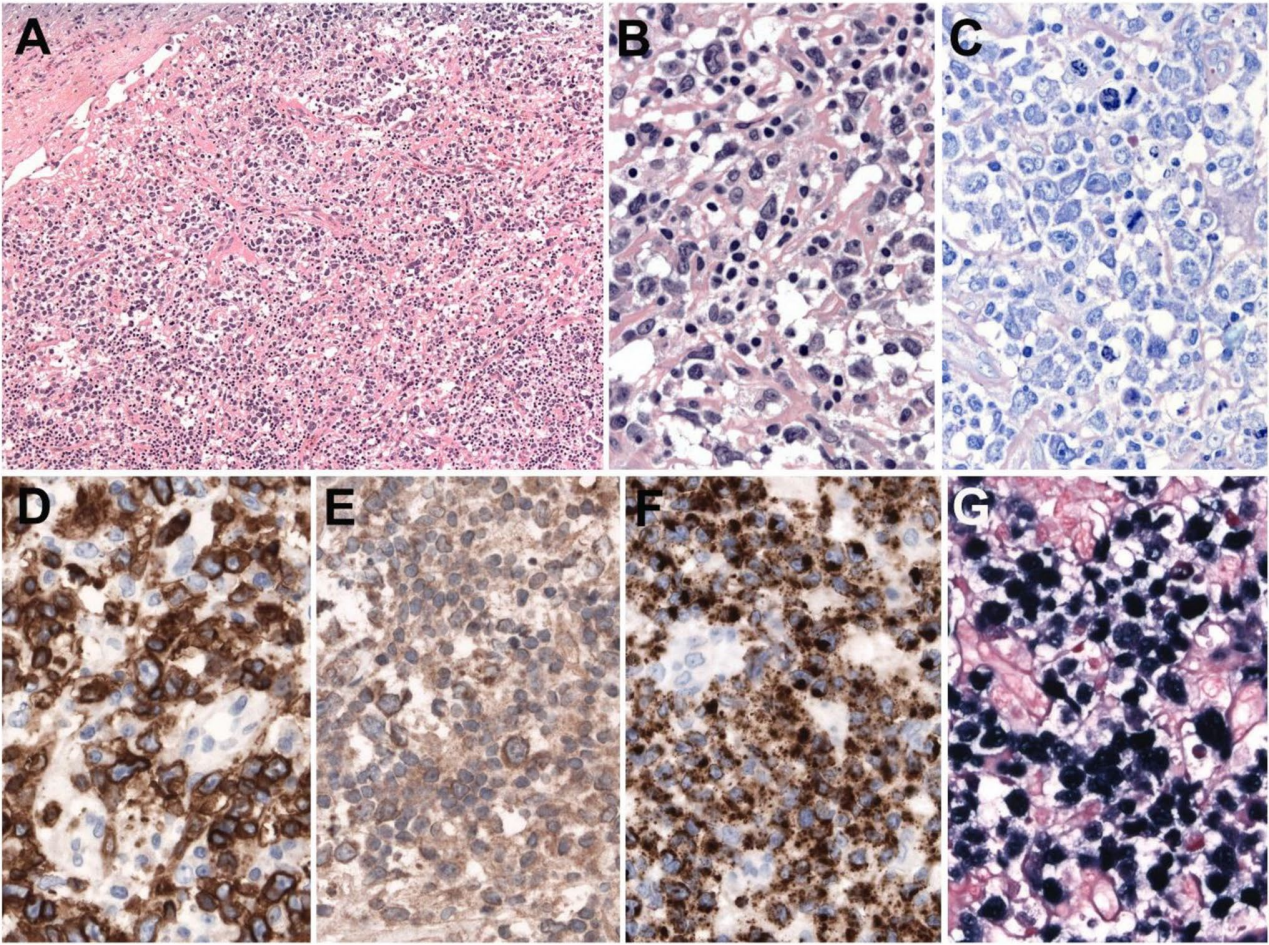
Primary nodal EBV+ T and NK cell lymphoma. **A** Complete effacement of the nodal architecture by a diffuse lymphoid infiltrate (original magnification ×200). **B** At higher magnification, there is a polymorphic infiltrate composed of large atypical cells with irregular nuclei, prominent nucleoli, and abundant cytoplasm admixed with small lymphocytes. **C** Giemsa stain demonstrates the atypical large cells with abundant mitosis. **D** The tumor cells are strongly CD3 positive. **E** TCR-gamma is positive as well as TIA1 (**F**). EBER in situ hybridization is positive in all infiltrating cells (**B**–**G**, original magnification ×400)

The lymph nodes show a relatively monomorphic infiltrate of atypical cells often without prominent angiocentricity or coagulative necrosis (Figure 7A-C). The tumor cells express CD3 and CD2 with lack of CD5 and CD4 expression (Figure 7D). TIA-1, granzyme B, and perforin are usually positive together with CD8 (>80%) (Figure 7F). CD56 might be positive in a minority of the cases (<20%) [58, 59]. CD30 and CD25 might be expressed raising the differential diagnosis with anaplastic large cell lymphoma (ALCL). TCR-ß is more often expressed than TCR-γ (43-64% vs 0-13%) (Figure 7E). EBER is positive in the majority of tumor cells (Figure 7G).

### Pathogenesis and molecular findings

Recent studies have demonstrated that this lymphoma is characterized by low genomic instability, upregulation of immune pathways (check point protein PD-L1) that promote immune evasion, and downregulation of EBV miRNAS [60]. The most common mutated genes are *TET2* (64%), *PIK3CD* (33%), *DDX3X* (20%), and *STAT3* (19%). A characteristic finding is recurrent losses of 14q11.2 where the TCR loci is supporting the T cell derivation of this lymphoma. [61]

## Immunodeficiency-associated lymphoproliferative disorders

The iatrogenic immunodeficiency-associated LPD include posttransplant LPD (PTLD) and the separately designated LPD arising in patients receiving methotrexate or other immunosuppressive agents, including those that may follow a variety of chemotherapeutic agents. They are included in this manuscript, as a significant subset are EBV+; however, as will be discussed, others which share many but not all features are not related to EBV infection. This discussion will concentrate on the PTLD.

The PTLD were recognized over half a century ago and are segregated from lymphomas and other iatrogenic immunodeficiency-related lymphoproliferative disorders (OIIA-LPD) in the 2001, 2008, and 2016/2017 WHO classifications and in the 2022 ICC [3]. The 2022 ICC retains the basic classification included in the revised 4th WHO classification, in large part because of major differences in clinical management from OIIA-LPD, which is consistent with recently published guidelines (Table 5) [62, 63]. The 2022 ICC does suggest that the OIIA-LPD, while segregated from the PTLD, should be categorized in a similar fashion. Like the PTLD, OIIA-LPD may be EBV+ or EBV-negative. Whether published clinical guidelines to handle PTLD are appropriate for patients with OIIA-LPDs is totally unknown,[62], [63] and some of the general guidance would be inappropriate in the non-transplant setting. The same applies to biologic studies of PTLD, such as studies of their molecular features or tumor microenvironmental characteristics. [64, 65, 66]

**Table 5:** Classification of posttransplant lymphoproliferative disorders

Non-destructive PTLDs
▪ Plasmacytic hyperplasia PTLD
▪ Infectious mononucleosis PTLD
▪ Florid follicular hyperplasia PTLD
Polymorphic PTLD^a^
Monomorphic PTLDs^b^
B cell neoplasms
▪ Diffuse large B cell lymphoma, NOS
▪ Burkitt lymphoma
▪ Multiple myeloma
▪ Plasmacytoma
▪ Other^c^
T cell neoplasms
▪ Peripheral T cell lymphoma, NOS
▪ Hepatosplenic T cell lymphoma
▪ Other
Classic Hodgkin lymphoma PTLD

*PTLD*, posttransplant lymphoproliferative disorder

^a^EBV-positive mucocutaneous ulcer which might resemble a polymorphic PTLD should be separately designated

^b^Classify according to lymphoma they resemble. The terminology for plasma cell myeloma has been updated to that used in the 2022 ICC

^c^Indolent small B cell lymphomas arising in transplant recipients are not included among the PTLDs, with the exception of EBV-positive extranodal marginal zone lymphoma of mucosa-associated lymphoid tissue (MALT lymphoma). See text for discussion related to EBV-negative marginal zone lymphomas

## Current approach to PTLD

The philosophy behind the 2022 ICC approach is when lymphoid proliferations are identified in patients following solid organ or stem cell transplantation, the first priority is to decide if it is a PTLD or whether it has some other explanation such as a specific infection, a non-specific inflammatory process, or if the transplanted organ is involved, whether it reflects rejection. In some cases, rejection and a PTLD may both be present. While many PTLD are EBV+, approximately 20–40% are not, with the proportion of late EBV-negative PTLD rising [62]. The EBV+ cases have a latency pattern III or less often pattern II. The presence of a small number of EBV+ cells is not equivalent to finding evidence of a PTLD, as scattered EBV+ cells can be seen in lymphoid proliferations even in immunocompetent hosts. Once the presence of a PTLD is confirmed, the second priority is to subclassify it. Excisional biopsy is recommended for diagnosis and, when possible, re-biopsy of recurrent lesions to rule out evolution or something other than a PTLD. [62, 63]

## Non-destructive PTLDs

The non-destructive PTLDs show underlying architectural preservation with a proliferation of predominantly small lymphocytes and polytypic plasma cells (plasmacytic hyperplasia PTLD); a lymphoplasmacytic proliferation with more prominent immunoblasts resembling infectious mononucleosis in an immune-competent host (infectious mononucleosis PTLD); or marked follicular hyperplasia (florid follicular hyperplasia PTLD). Because these are histologically non-specific reactive proliferations, oftentimes, they can only be diagnosed with extensive EBV positivity, best seen with EBER in situ hybridization, or less often because they are clearly mass-forming. Some of these patients may have synchronous or metachronous overt PTLD at other sites. The non-destructive PTLD may demonstrate clonal populations with cytogenetic and mutational abnormalities, although most are non-clonal. [13, 64, 65]

## Polymorphic PTLD

The most classic but problematic type of PTLD is polymorphic PTLD, which causes architectural effacement of underlying tissues and includes variably sized and shaped lymphoid cells, plasma cells, and immunoblasts that may closely resemble Reed–Sternberg cells. Angioinvasion and geographic areas of necrosis may be present. They are not supposed to fulfill the criteria for a lymphoma in an immunocompetent host, a criterion that is not always applied. Polymorphic PTLDs usually demonstrate clonal B cell populations, although the clone size may be small, and they may demonstrate mutations although reportedly not as often as with monomorphic PTLD [67]. The published literature is not consistent in terms of whether patients with polymorphic PTLD have a better prognosis than those with monomorphic PTLD with a recent large study reporting similar overall survivals (see below). [68]

## Monomorphic PTLDs

Monomorphic PTLDs are the most frequent type of PTLD and need to be further classified based on the lymphoma, which they most closely resemble. Although sometimes considered to be proliferations of monomorphic large transformed cells/immunoblasts, monomorphic today really refers to these cases resembling varied lymphomas in immune-competent hosts, not all of which are sheets of large cells or even totally monomorphic. Some cases are polymorphic-appearing, including some B cell monomorphic PTLD that may have plasmacytic differentiation or a high content of reactive T cells, some may have predominantly plasma cells, and many of the monomorphic T cell PTLD are not composed of sheets of large transformed cells. Although traditionally not including cases that resemble one of the small B cell lymphomas, the 2016/2017 WHO revision included EBV+ MALT lymphomas as a type of PTLD. A more recent study has proposed that EBV-negative marginal zone lymphomas, which usually are of MALT type and often in the gastrointestinal tract, also be included as a form of monomorphic PTLD [69]. The most common of the monomorphic PTLDs resemble DLBCL. They are often of non-germinal center B cell type, with the latter particularly true for EBV+ cases. Although some do report significant mutational and gene expression profiling differences between monomorphic PTLD of DLBCL-type and similar lymphomas in immune-competent hosts, others emphasize their similarities at least for the EBV-negative cases [64, 67, 70]. In one study, it was pointed out that “In contrast to B cell PTLDs, the molecular and genomic alterations observed in T/NK-PTLD appear similar to those reported for peripheral T cell lymphomas occurring in immunocompetent hosts,…” [71] Monomorphic B cell PTLD of Burkitt lymphoma type is an important diagnosis because these patients are more likely to need prompt aggressive therapies [72]. Cases of what is now known as large B cell (formerly Burkitt-like) lymphoma with 11q aberration also have been specifically recognized in the posttransplant setting [73]. Monomorphic PTLD may also resemble plasma cell neoplasms, and it is important then to distinguish those that are plasmacytoma-like, which may do well even without aggressive therapies, from the typically more aggressive cases that resemble multiple myeloma. With the exception of cases that resemble T cell large granular lymphocytic leukemias, monomorphic T cell PTLD that may resemble many different types of T/NK cell neoplasms are typically very aggressive and much less likely to be EBV+.

## Classic Hodgkin lymphoma PTLD

Although not common in the posttransplant setting, some PTLD closely resemble classic Hodgkin lymphoma (CHL) and are almost always EBV+. Caution is advised since many other PTLD have cells resembling Reed–Sternberg cells, but this is an important specific diagnosis to make, since it is another form of PTLD typically treated promptly with standard CHL-therapy. Immunohistologic studies are critical in excluding a monomorphic or even polymorphic PTLD. In a recent large single-institution study, CHL PTLD had the best prognosis of all the destructive type PTLD with a 2-year point estimate overall survival of 1.00 versus monomorphic B cell PTLD (0.62) and polymorphic PTLD (0.69). [68] This is in contrast to what has been reported in OIIA-LPD in patients with rheumatic diseases where overall survival of Hodgkin-type LPD was similar to DLBCL-type, but PFS was worse for the Hodgkin cases. [74]

## Conclusions

The better understanding of the EBV-associated LPDs has resulted in refining the diagnostic criteria of well-defined entities and recognition of new entities. The diagnosis and treatment of these diseases are complex and require a multiparameter approach incorporating detailed clinical information together with histologic and immunophenotypic features.
